# A Computational Study of the Mechanism of Succinimide Formation in the Asn–His Sequence: Intramolecular Catalysis by the His Side Chain

**DOI:** 10.3390/molecules21030327

**Published:** 2016-03-09

**Authors:** Ohgi Takahashi, Noriyoshi Manabe, Ryota Kirikoshi

**Affiliations:** Faculty of Pharmaceutical Sciences, Tohoku Pharmaceutical University, 4-4-1 Komatsushima, Aoba-ku, Sendai 981-8558, Japan; manabe@tohoku-pharm.ac.jp (N.M.); kirikoshi@tohoku-pharm.ac.jp (R.K.)

**Keywords:** asparagine residue, deamidation, succinimide, nonenzymatic reaction, Asn–His sequence, intramolecular catalysis, histidine imidazole group, proton-transfer mediator, computational chemistry, density functional theory

## Abstract

The rates of deamidation reactions of asparagine (Asn) residues which occur spontaneously and nonenzymatically in peptides and proteins via the succinimide intermediate are known to be strongly dependent on the nature of the following residue on the carboxyl side (Xxx). The formation of the succinimide intermediate is by far the fastest when Xxx is glycine (Gly), the smallest amino acid residue, while extremely slow when Xxx is bulky such as isoleucine (Ile) and valine (Val). In this respect, it is very interesting to note that the succinimide formation is definitely accelerated when Xxx is histidine (His) despite its large size. In this paper, we computationally show that, in an Asn–His sequence, the His side-chain imidazole group (in the neutral Nε-protonated form) can specifically catalyze the formation of the tetrahedral intermediate in the succinimide formation by mediating a proton transfer. The calculations were performed for Ace−Asn−His−Nme (Ace = acetyl, Nme = methylamino) as a model compound by the density functional theory with the B3LYP functional and the 6-31+G(d,p) basis set. We also show that the tetrahedral intermediate, once protonated at the NH_2_ group, easily releases an ammonia molecule to give the succinimide species.

## 1. Introduction

**Scheme 1:**
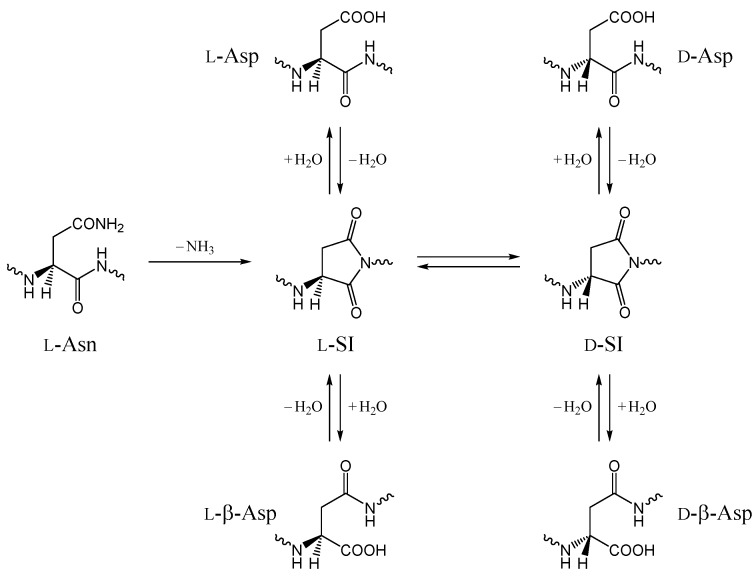
Succinimide (SI)-mediated nonenzymatic reactions of Asn and Asp residues.

Succinimide (SI)-mediated reactions of asparagine (Asn) and aspartic acid (Asp) residues in peptides and proteins (Scheme 1) have been extensively studied in many fields of chemical, biological, and pharmaceutical sciences [1,2,3,4,5,6,7,8,9,10,11,12,13,14,15,16,17,18,19,20,21,22,23,24,25,26,27,28,29,30,31,32,33,34,35,36,37,38]. These reactions occur spontaneously and nonenzymatically both *in vivo* and *in vitro*, and produce biologically uncommon l-β-Asp, d-Asp, and d-β-Asp residues. In fact, these reactions may lead to protein degradations and, hence, to aging and pathologies [9,15,20,21,22,23,26,32,33,37,38]. In the case of Asn residues, the SI formation results in deamidation. Release of an ammonia molecule makes Asn deamidation reactions irreversible. The SI (l-SI) intermediate formed from an l-Asn residue can undergo hydrolysis either to an l-Asp or an l-β-Asp residue, typically in a ratio of 1:3 for peptides [1,2,3,4,5,7,8,13]. The d-Asp and d-β-Asp residues may also be formed because the SI intermediate is racemization-prone [39,40,41]. Since the rate of Asn deamidation varies widely depending on the peptide and protein structures, it has been proposed that this reaction functions as a molecular clock by regulating the timing of biological events such as protein turnover [18,25].

**Scheme 2:**
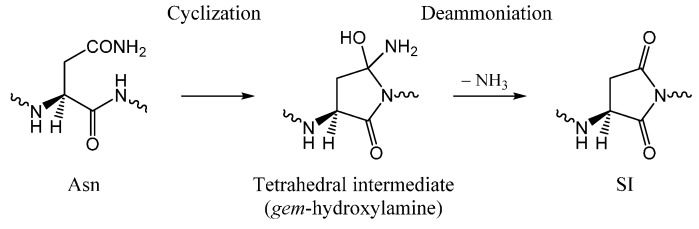
Two-step (cyclization-deammoniation) mechanism of succinimide (SI) formation from an Asn residue.

The SI formation from Asn residues has been regarded as an intramolecular nucleophilic substitution reaction occurring in two steps (cyclization-deammoniation) (Scheme 2) [42,43,44]. In the first step, the peptide-bond nitrogen of the following residue on the carboxyl side (Xxx), also commonly referred to as the *n* + 1 residue, attacks the Asn side-chain amide carbon to form a five-membered ring tetrahedral intermediate. In the second step, an NH_3_ molecule is released from the intermediate to give the SI species. Proton transfers have to occur concomitantly with the bond formation or cleavage in these steps; however, detailed proton transfer mechanisms have not been elucidated. Our recent calculations about SI formations from Asn and Asp residues suggest that proton transfers mediated by a carboxylic acid molecule may occur in acidic conditions [44,45]. It should also be noted that the SI-mediated mechanism of Asn deamidation, which involves the nucleophilic attack by the main-chain nitrogen of the following residue, is not applicable to free Asn. In this regard, it is interesting to note that free glutamine (Gln) may deamidate via another five-membered ring intermediate, 5-oxoproline, which results from nucleophilic attack by the Gln main-chain nitrogen on the side-chain amide carbon [46].

It has been well known that the rates of SI formation from Asn residues are strongly dependent on the nature, especially size, of the following residue Xxx [5,7,8,16,17,18,19,24,25]. The formation of the SI intermediate is by far the fastest when Xxx is glycine (Gly), the smallest amino acid residue, while extremely slow when Xxx is bulky, such as isoleucine (Ile) and valine (Val). In this respect, it is very interesting to note that the SI formation is definitely accelerated when Xxx is histidine (His) despite its large size [7,16,17,18,19,24,25]. For example, deamidation in pentapeptide Gly–Gly–Asn–His–Gly is 2.3 and 1.3 times faster than in Gly–Gly–Asn–Ala–Gly (Ala = alanine) and Gly–Gly–Asn–Ser–Gly (Ser = serine), respectively (Tris-HCl buffer, pH 7.4, 37 °C) [18]. Indeed, on average, Asn deamidation reactions seem to be fastest when Xxx = His except for when Xxx = Gly. Goolcharran *et al.* [17] investigated pH-dependence of deamidation in a pentapeptide Gly–Gln–Asn–His–His, and showed that the *n* + 1 His residue acts as a catalyst at all pH values investigated (pH 5–10). Since p*K*_a_ of the doubly protonated cationic form of the His side chain (imidazole ring) is around 6, the His residue is expected to exist mostly in the neutral state (equilibrium between the Nδ- and Nε-protonated forms) at physiological pH.

**Figure 1:**
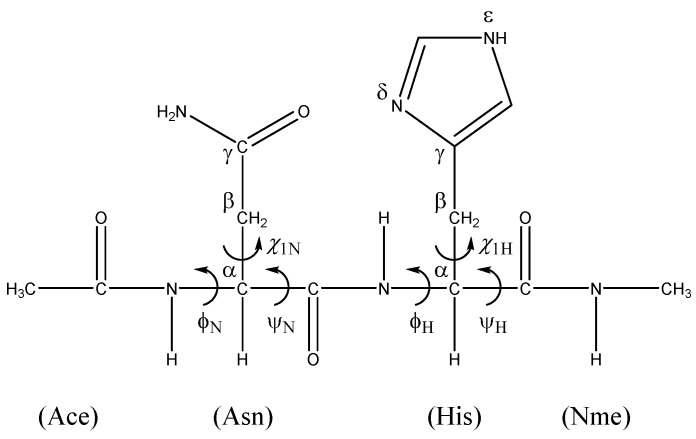
The model compound (Ace–Asn–His–Nme) used in the present study. Both the Asn and His residues are in the l-configuration. The φ_N_ (C–N–C_α_–C) and ψ_N_ (N–C_α_–C–N) dihedral angles characterize the main-chain conformation of the Asn residue, and φ_H_ and ψ_H_ are the corresponding dihedral angles of the His residue. The χ_1N_ and χ_1H_ dihedral angles (N–C_α_–C_β_–C_γ_) are for the side chains of the Asn and His residues, respectively.

In this paper, a possible reaction mechanism is computationally shown for SI formation in an Asn–His sequence, where the side-chain imidazole group (in its neutral Nε-protonated form) catalyzes the formation of the tetrahedral intermediate. The calculations were performed by the density functional theory (DFT) for a model compound Ace–Asn–His–Nme (Figure 1), where Ace and Nme stand for acetyl and methylamino groups, respectively. In the proposed mechanism, abstraction of the His main-chain NH proton by the Nδ atom and subsequent transfer of the abstracted proton to the Asn side-chain oxygen atom occur in the first cyclization step. It is also shown that the intermediate, once protonated at the NH_2_ group, readily releases an NH_3_ molecule to form the SI species.

## 2. Results and Discussion

**Figure 2:**
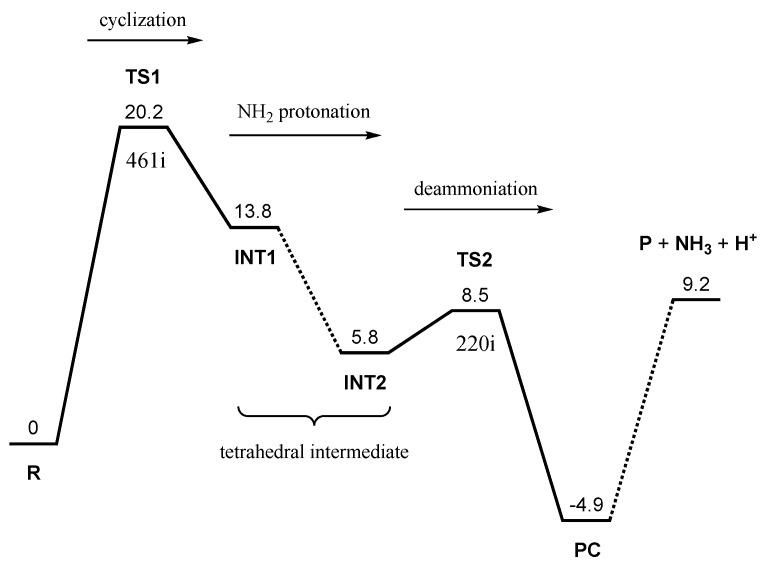
Energy profile for the SI formation from the model compound shown in Figure 1. ZPE- and hydration free energy-corrected relative energies are shown in kcal·mol^−1^. R: reactant; TS: transition state; INT: intermediate; P: product; PC: product complex. The imaginary frequency (cm^−1^) is also shown for TS1 and TS2.

**Figure 3:**
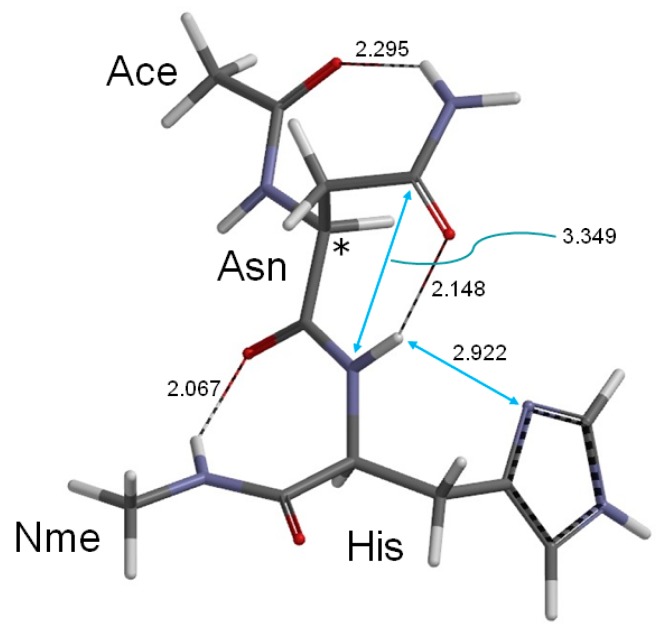
The optimized geometry of the reactant R (model compound, Figure 1) (φ_N_ = −167°, ψ_N_ = −178°, χ_1N_ = −138°, φ_H_ = −88°, ψ_H_ = 64°, χ_1H_ = −73°). Relevant interatomic distances are shown in Å. The α carbon of Asn is indicated by an asterisk.

**Figure 4:**
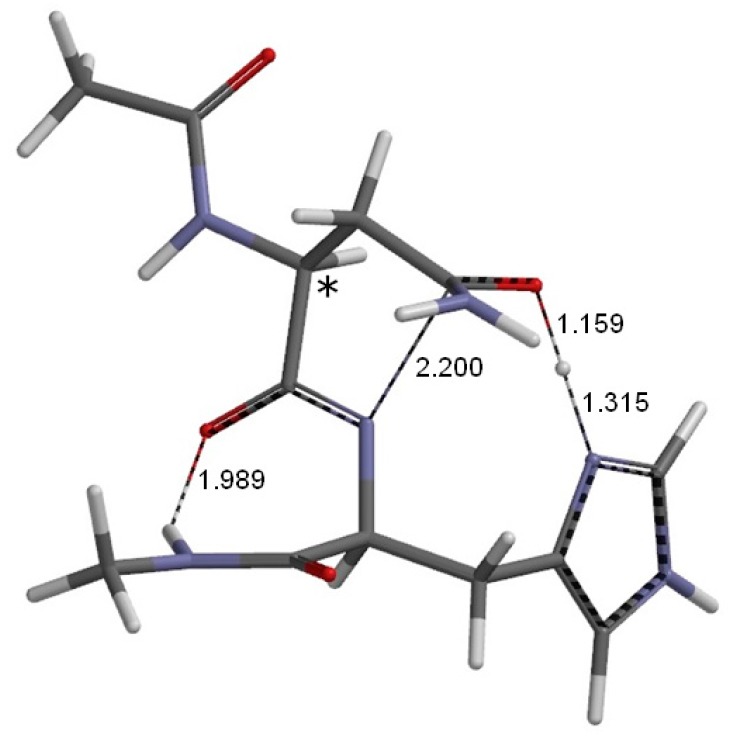
The optimized geometry of TS1, the transition state of the first step (cyclization) (φ_N_ = −169°, ψ_N_ = −158°, χ_1N_ = 170°, φ_H_ = −83°, ψ_H_ = 78°, χ_1H_ = −59°). Relevant interatomic distances are shown in Å. The asterisked carbon corresponds to the Asn α carbon in the reactant R.

**Figure 5:**
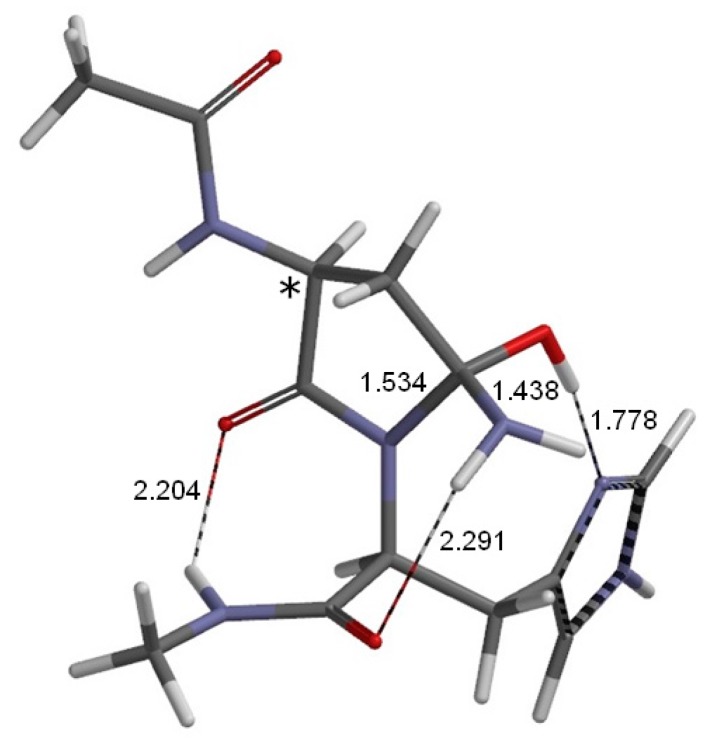
The optimized geometry of INT1, the intermediate directly connected to TS1 (φ_N_ = −169°, ψ_N_ = −150°, χ_1N_ = 155°, φ_H_ = −85°, ψ_H_ = 85°, χ_1H_ = −65°). Relevant bond distances are shown in Å. The asterisked carbon corresponds to the Asn α carbon in the reactant R.

**Figure 6:**
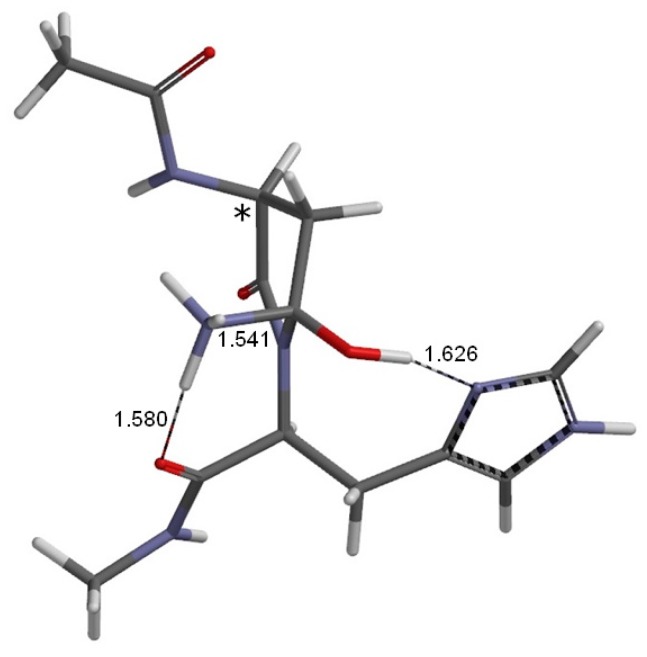
The optimized geometry of INT2, the protonated intermediate directly connected to TS2 (φ_N_ = −174°, ψ_N_ = −105°, χ_1N_ = 88°, φ_H_ = −95°, ψ_H_ = 144°, χ_1H_ = −54°). Relevant bond distances are shown in Å. The asterisked carbon corresponds to the Asn α carbon in the reactant R.

**Figure 7:**
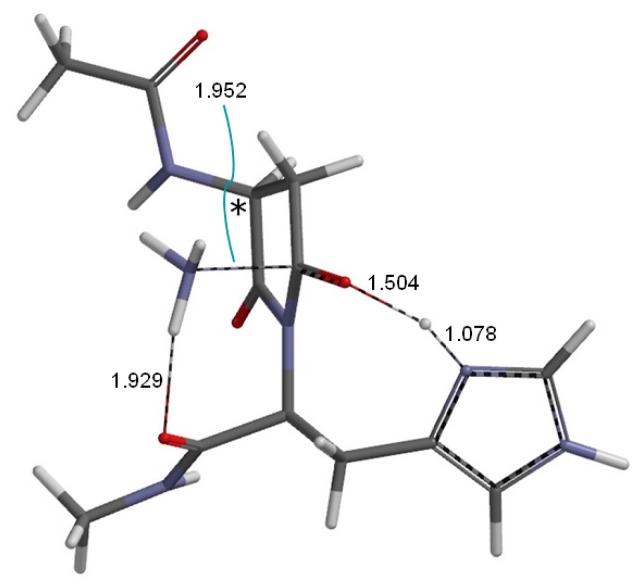
The optimized geometry of TS2, the transition state of the second step (deammoniation from the protonated intermediate, INT2) (φ_N_ = −174°, ψ_N_ = −116°, χ_1N_ = 103°, φ_H_ = −74°, ψ_H_ = 112°, χ_1H_ = −62°). Relevant interatomic distances are shown in Å. The asterisked carbon corresponds to the Asn α carbon in the reactant R.

**Figure 8:**
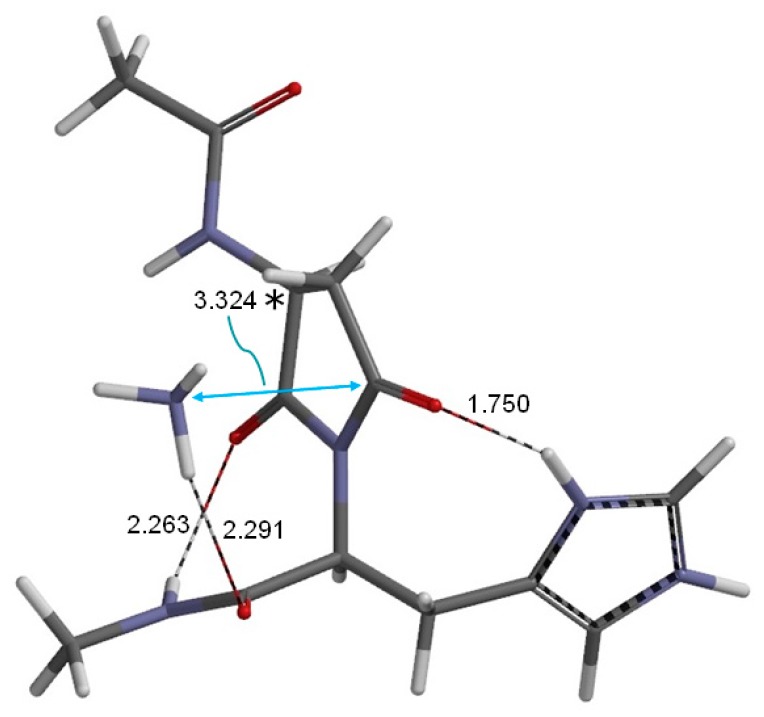
The optimized geometry of the product complex PC (complex between the SI product and an NH_3_ molecule) (φ_N_ = −163°, ψ_N_ = −147°, χ_1N_ = 146°, φ_H_ = −80°, ψ_H_ = 91°, χ_1H_ = −56°). The imidazole ring is in the cationic form. Relevant interatomic distances are shown in Å. The asterisked carbon corresponds to the Asn α carbon in the reactant R.

**Figure 9:**
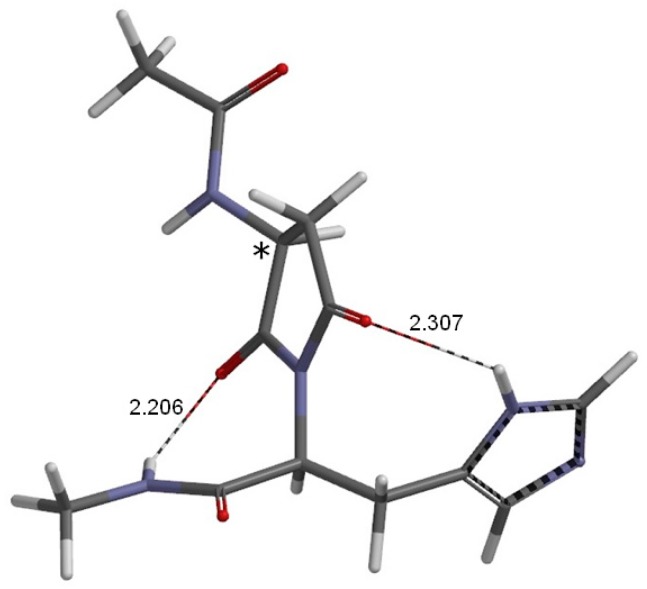
The optimized geometry of the SI product P (φ_N_ = −172°, ψ_N_ = −145°, χ_1N_ = 140°, φ_H_ = −90°, ψ_H_ = 71°, χ_1H_ = −53°). The imidazole ring is in a neutral form. Hydrogen bond distances are shown in Å. The asterisked carbon corresponds to the Asn α carbon in the reactant R.

Figure 2 shows the energy profile obtained from the present calculations, and Figure 3, Figure 4, Figure 5, Figure 6, Figure 7, Figure 8 and Figure 9 show optimized geometries. Geometry optimizations were performed by using the B3LYP functional and the 6-31+G(d,p) basis set, and relative energies were corrected for the zero-point energy (ZPE) and the hydration free energy calculated by the SM8 (solvation model 8) continuum model [47,48]. The reactant, transition state, intermediate, and product are abbreviated as R, TS, INT, and P, respectively, and PC stands for the product complex formed between P, an NH_3_ molecule, and a proton (see below). The second step (deammoniation) was calculated for the NH_2_-protonated form of the intermediate (INT2). Therefore, in order to compare energies between the first and the second steps, an experimental free energy of hydration was added to the energies of the first-step geometries (R, TS1, and INT1) (see below for more details). The Cartesian coordinates, total energies, ZPEs, and SM8 hydration free energies of the optimized geometries are provided in the Supplementary Materials.

Figure 3 shows the optimized geometry for the reactant R, *i.e.*, the model compound. In this geometry, the Asn residue is in an extended conformation (φ_N_ = −167°, ψ_N_ = −178°) while φ_H_ = −88° and ψ_H_ = 64°. This conformation enables the His imidazole ring to participate in the reaction. As described below, a somewhat complicated process to the neutral tetrahedral intermediate (INT1) proceeds in a single step from this reactant conformer via the transition state TS1 (Figure 4).

In R, the NH hydrogen of the His main chain forms a hydrogen bond (2.148 Å) to the amide oxygen of the Asn side chain; therefore, the distance between the His main-chain nitrogen and the amide carbon of the Asn side chain (the two atoms to be bonded) is as long as 3.349 Å. In the initial stage of the first step, proton transfer occurs from the His main-chain NH toward the His Nδ atom to form a cationic form of the imidazole ring (the initial distance between the NH hydrogen and His Nδ atoms is 2.922 Å). The proton-transferred state corresponds to a very flat region on the potential energy surface, but no energy minimum was found in this region. This proton transfer (abstraction of the NH proton) enhances the nucleophilicity of the main-chain N atom, and induces nucleophilic attack of this nitrogen on the Asn side-chain amide carbon. This attack results in cyclization to a five-membered ring. Concomitantly with this N–C bond formation, proton transfer occurs from the His Nδ to the Asn side-chain amide oxygen, resulting in a *gem*-hydroxylamine tetrahedral intermediate (INT1, Figure 5). At TS1, the distance of the forming N–C bond is 2.200 Å, and the transferring proton is closer to the oxygen atom than to the Nδ atom (see Figure 4).

These changes occur in a single step via TS1, as revealed by intrinsic reaction coordinate (IRC) calculations followed by full geometry optimizations, and this first step is predicted to be rate-determining in the SI formation. The activation barrier (*i.e.*, the energy difference between R and TS1) is 20.2 kcal·mol^−1^ (after ZPE and hydration free energy correction). Considering that typical experimental values for activation energies of Asn deamidation reactions are 20–24 kcal·mol^−1^ [1,4,5,36], we may say that the His imidazole ring (neutral form) can catalyze SI formation in the Asn–His sequence by acting as a proton-transfer mediator. It should be noted that the Asn main-chain carboxyl oxygen forms a hydrogen bond to the NH hydrogen of Nme throughout the first step (the distances are 2.067, 1.989, and 2.204 Å in R, TS1, and INT1, respectively). This hydrogen bond is important in placing the His side chain in the right position to catalyze the cyclization as a proton-transfer mediator. Upon cyclization, a new hydrogen bond (2.291 Å) is formed between the NH_2_ group and the His oxygen. The energy of INT1 relative to R is 13.8 kcal·mol^−1^.

The second step (NH_3_ release) was calculated for the NH_2_-protonated form of the tetrahedral intermediate (INT2). This is based on the p*K*_a_ value of 9.96 for 1-amino-1-propanol (conjugate acid) [49]. Although this is only one example which we found in the literature for p*K*_a_ values of *gem*-hydroxylamine species, it is fully expected that the NH_2_ group on the five-membered ring of INT1 is rapidly protonated at neutral or physiological pH.

Figure 6 shows the optimized geometry of INT2. To compare the energies of the neutral and protonated forms of the intermediate, INT1 and INT2, the hydration free energy of proton (H^+^) has to be added to the energy of the former (note that the electronic energy of a bare proton is zero). We used an experimental value of −265.9 kcal·mol^−1^ [50] for the hydration free energy of proton. Thus, the energy profile shown in Figure 2 was obtained by adding this value for R, TS1, and INT1. As seen from Figure 2, the intermediate was calculated to be stabilized by 8 kcal·mol^−1^ upon protonation, so that the energy of INT2 relative to R is 5.8 kcal·mol^−1^. Moreover, there are noticeable geometrical differences between INT1 and INT2 as may be seen from the dihedral angle values shown in the captions of Figure 5 and Figure 6. It seems that this is mainly due to changes in hydrogen bonds. The hydrogen bond involving Nme is broken in INT2. Instead, the hydrogen bond involving the His oxygen has been highly strengthened upon protonation (from 2.291 Å to 1.580 Å) because of the positive charge of the NH_3_^+^ group.

From INT2, an NH_3_ molecule is released via the transition state TS2 (Figure 7). The local activation barrier of this process is only 2.7 kcal·mol^−1^. Concomitantly with the C–N bond cleavage, proton transfer occurs from the OH group to the His Nδ atom producing an SI ring and a cationic form of the imidazole ring. The geometry of the resulting product complex (PC) is shown in Figure 8. In TS2, the distance of the cleaving C–N bond is 1.952 Å; the corresponding distances in INT2 and PC are 1.541 and 3.324 Å, respectively. The proton transfer is almost completed in TS2 (the N–H distance is 1.078 Å). In PC, the released NH_3_ molecule is hydrogen-bonded to the His oxygen (2.291 Å) and the NH hydrogen of Nme again forms a hydrogen bond (2.263 Å) to one of the SI oxygens. The other SI oxygen forms a hydrogen bond (1.750 Å) to the cationic imidazole ring. PC was calculated to be more stable than R by 4.9 kcal·mol^−1^.

The geometry of the SI product (P) shown in Figure 9 was obtained by removing the NH_3_ molecule and the proton bound to the His Nε atom from PC and optimizing the remaining part. While geometrical change in this optimization is small, the separated state (P + NH_3_ + H^+^) is higher in energy than PC by about 14 kcal·mol^−1^ (9.2 kcal·mol^−1^ relative to R). Here, the energy of “H^+^” is the experimental hydration free energy of proton (see above), corresponding to a hydrated H_3_O^+^ ion. Although part of this large value may be attributed to basis-set superposition error, the separated products will be entropically favored. Indeed, SI formation from Asp residues was shown to be entropy-driven for short peptides [13]. Therefore, the Asn reactant state and the SI product state are expected to be comparable in free energy.

## 3. Computational Methods

Figure 1 shows the model compound used in the present study, in which an Asn–His sequence is capped with Ace and Nme groups on the *N*- and *C*-termini, respectively. All calculations were performed using Spartan’14 [51]. As in our previous studies [40,41,44,45], energy-minimum and transition state geometries were located in vacuum without any constraints by DFT, with the B3LYP functional and the 6-31+G(d,p) basis set. Vibrational frequency calculations were performed for all of the optimized geometries to confirm them as energy minima (with no imaginary frequency) or transition states (with a single imaginary frequency) and to correct the relative energies for ZPE. IRC calculations were performed from the transition states followed by full geometry optimizations to confirm that each transition state connects two energy minima, as shown in Figure 2. Furthermore, hydration effects have been included by single-point calculations at the same level of theory employing the SM8 continuum model [47,48].

## 4. Conclusions

The present calculations have shown that succinimide formation in an Asn–His sequence can be specifically catalyzed intramolecularly by the His imidazole ring (in its Nε-protonated form). The catalytic effect of the imidazole ring operates in the first step of the cyclization-deammoniation mechanism. In the very initial stage of the reaction, the His main-chain NH proton is abstracted by the His Nδ atom. This enhances the nucleophilicity of the His main-chain nitrogen, which then attacks the amide carbon of the Asn side chain resulting in cyclization. Proton transfer from the His Nδ atom to the Asn side-chain oxygen occurs concomitantly with this cyclization. It is interesting to note that this complicated process to form the five-membered ring tetrahedral intermediate occurs in a single step. The intermediate, which is a *gem*-hydroxylamine species, is expected to be rapidly protonated at the NH_2_ group. We have shown that the protonated intermediate can easily undergo deammoniation resulting in the succinimide product.

## Supplementary Materials

The following are available online at: http://www.mdpi.com/1420-3049/21/3/327/s1, the Cartesian coordinates, total energies, zero-point energies, and SM8 hydration free energies of the optimized geometries.

## Abbreviations

The following abbreviations are used in this manuscript:

DFT: density functional theory
INT: intermediate
IRC: intrinsic reaction coordinate
P: product
PC: product complex
R: reactant
SI: succinimide
TS: transition state
ZPE: zero-point energy
