# Supplementary material for: A Computational Study of the Mechanism of Succinimide Formation in the Asn–His Sequence: Intramolecular Catalysis by the His Side Chain
Source: Molecules. 2016 Mar 9;21(3):327. doi: 10.3390/molecules21030327 (PMC6274526; doi:10.3390/molecules21030327)
Supplement: Supplementary file 1 [file molecules-21-00327-s001.pdf]

Supplementary Materials: A Computational Study of the Mechanism of Succinimide Formation in the Asn–His Sequence: Intramolecular Catalysis by the His Side Chain

Ohgi Takahashi, Noriyoshi Manabe and Ryota Kirikoshi

Cartesian coordinates (in Å), total energies (in au), zero-point energies (in kJ mol^{-1}), and SM8 hydration free energies (in kJ mol^{-1}) of the B3LYP/6-31+G(d,p) optimized geometries.

Geometry: R

Total energy: −1136.999684
Zero-point energy: 920.3961
Hydration free energy: −94.4271731

Cartesian coordinates:

6	−5.023267737	0.347721433	2.158811172
6	−4.075648744	−0.102801669	1.064569563
8	−4.403945115	−0.958843962	0.232100541
7	−2.855529810	0.492231680	1.039447469
1	−2.541298583	1.109665462	1.780998167
6	−1.829224048	0.065632579	0.108341611
6	−0.495749007	0.689480417	0.549593118
8	−0.428379550	1.380061854	1.581142910
7	0.555529818	0.425442051	−0.241806909
1	0.410518212	−0.138217688	−1.080244826
6	1.938426088	0.739316795	0.136634615
6	−2.192681021	0.408303338	−1.371665927
6	−1.829924336	−0.766483816	−2.268552478
8	−0.704332676	−0.882692624	−2.758698735
7	−2.819502778	−1.682084281	−2.431690994
1	−2.582058313	−2.553362228	−2.885429023
6	2.325383663	2.183944444	−0.278600444
8	3.217148218	2.424190530	−1.090723167
7	1.613643182	3.150885343	0.351976745
1	0.888977099	2.864321436	1.003345851
6	1.829112620	4.561663223	0.068544193
6	2.899836906	−0.295962103	−0.467686257
6	2.843823508	−1.636237206	0.205439633
6	3.866319029	−2.297299185	0.848575958
7	3.321894522	−3.488332044	1.288969829
6	2.012163427	−3.499761631	0.902785334
7	1.691588268	−2.402676509	0.249638658
1	−1.714926594	−1.023464443	0.195170614
1	1.984023024	0.690724603	1.231153525
1	−1.645741994	1.288817185	−1.715496396
1	−3.261716509	0.616174445	−1.427101830
1	3.912477175	0.108729863	−0.401568401
1	2.689223147	−0.394213041	−1.539916028
1	4.899458642	−2.036599377	1.021150414
1	3.805175772	−4.216282959	1.793342812
1	1.349224315	−4.325949345	1.119202071
1	−5.948848476	0.705389160	1.699339278
1	−4.607314411	1.135125460	2.792420555
1	−5.277197589	−0.516593418	2.779470300
1	1.190572173	5.151412841	0.729154410
1	2.874691218	4.835724911	0.239887263
1	1.587038356	4.800694939	−0.972817927
1	−3.620988177	−1.657187580	−1.809117328

Geometry: TS1

Total energy: −1136.953376
Zero-point energy: 906.8741
Hydration free energy: −117.8530274

Cartesian coordinates:

6	5.534172005	0.597853581	1.091921339
6	4.262741491	1.085799949	0.416154931
8	4.241739460	2.126792055	−0.244197406
7	3.166787893	0.292310917	0.565502776
1	3.150017486	−0.450408836	1.258980139
6	1.841118696	0.687789404	0.130157462
6	0.820982568	−0.316201345	0.701923333
8	1.125204193	−1.027994285	1.701622320
7	−0.295252741	−0.303212359	−0.000553835
1	−1.331472606	1.926152616	−0.874131337
6	−1.398353007	−1.153003701	0.423680793
6	1.672629845	0.706505046	−1.399195647
6	0.221621011	0.882570973	−1.780419135
8	−0.367838204	2.004884547	−1.513800529
7	−0.245403317	0.151557885	−2.825061042
1	−1.200381164	0.320668409	−3.110745784
1	0.074994511	−0.801675874	−2.925862536
6	−1.185193546	−2.589140490	−0.109173746
8	−1.766701978	−3.020373943	−1.111053298
7	−0.287147372	−3.308223358	0.609296059
1	0.280076774	−2.777228857	1.273892703
6	0.163378240	−4.619959501	0.173956486
6	−2.746067509	−0.609079984	−0.090181050
6	−3.054336989	0.777183920	0.380634883
6	−3.996776448	1.201344528	1.285363910
7	−3.868279325	2.576811091	1.370911694
6	−2.875297446	2.958682015	0.537446909
7	−2.373288869	1.897058060	−0.071514011
1	1.612716657	1.690433906	0.516121177
1	−1.424889987	−1.196347580	1.523749043
1	2.061120432	−0.227795129	−1.817236585
1	2.237458688	1.539347378	−1.831262046
1	−3.546862045	−1.279439772	0.235774457
1	−2.747658490	−0.647020127	−1.183871914
1	−4.729127127	0.655977579	1.859156540
1	−4.414182297	3.191691589	1.956307304
1	−2.548656333	3.978873767	0.403105402
1	6.304937357	0.455092329	0.328963286
1	5.403061396	−0.334996537	1.647319858
1	5.888383605	1.376390041	1.773868790
1	0.590640139	−5.152554028	1.027011656
1	−0.686130421	−5.183786846	−0.216490985
1	0.921098314	−4.556023034	−0.618912156

Geometry: INT1

Total energy: −1136.973842
Zero-point energy: 921.2655
Hydration free energy: −105.3594879

Cartesian coordinates:

6	5.630716824	0.749923190	1.104356899
6	4.379963045	1.183570747	0.357710882
8	4.384422158	2.133288780	−0.421900753
7	3.256365916	0.440015656	0.592832477
1	3.230768080	−0.188160340	1.387395659
6	1.960527981	0.843157210	0.080968061
6	0.878494712	−0.010816093	0.733476109
8	0.974738329	−0.554084618	1.842065093
7	−0.163677468	−0.137179081	−0.130031407
1	−1.255378471	1.867604183	−1.127602189
6	−1.277950870	−1.023302958	0.253254690
6	1.683441752	0.611080540	−1.409986250
6	0.147396709	0.509158776	−1.485905001
8	−0.358760833	1.808451424	−1.545341841
7	−0.309966235	−0.254701962	−2.615447693
1	−1.175917095	0.112629171	−2.989765602
1	−0.376706654	−1.257376444	−2.467489200
6	−0.924242972	−2.474444500	−0.155629170
8	−1.279470396	−2.962562661	−1.231277517
7	−0.181612251	−3.142385692	0.761430776
1	0.208101475	−2.595463983	1.523440630
6	0.354545254	−4.469806261	0.492149128
6	−2.680201817	−0.642909496	−0.263002551
6	−3.215756954	0.648674071	0.275998978
6	−4.186043604	0.853519133	1.229439735
7	−4.307777967	2.224846149	1.352052842
6	−3.425275810	2.795115369	0.486957492
7	−2.757514993	1.872071024	−0.175633170
1	1.778153812	1.899720892	0.319709442
1	−1.299482420	−0.977115257	1.346494258
1	2.129225009	−0.335964887	−1.730768089
1	2.038461771	1.414425521	−2.054272457
1	−3.348633575	−1.455448766	0.039695072
1	−2.696357721	−0.637555977	−1.355083417
1	−4.785848262	0.167899719	1.807906968
1	−4.939538030	2.715350960	1.967545477
1	−3.309967070	3.863845385	0.377586342
1	6.417109146	0.535535385	0.375521222
1	5.480956937	−0.129006803	1.737850504
1	5.974930387	1.583806677	1.723184695
1	0.638285301	−4.936309053	1.437730805
1	−0.410798914	−5.075622839	0.003882132
1	1.230797719	−4.429717977	−0.166482906

Geometry: INT2

Total energy: −1137.371933
Zero-point energy: 958.9732
Hydration free energy: −243.9719846

Cartesian coordinates:

6	5.550528947	−0.071522073	0.518343334
6	4.324770853	0.781497984	0.274822080
8	4.371173924	1.920544493	−0.160347598
7	3.095979087	0.175811484	0.570871870
1	3.149704720	−0.586787672	1.240308347
6	1.914222716	1.040884504	0.735353500
6	0.763610898	0.137740996	1.198362791
8	0.646128444	−0.367066428	2.300612057
7	−0.096892512	−0.067561556	0.129440641
1	−1.227433858	1.561419881	−1.518369282
6	−1.249451878	−0.962101052	0.310673724
6	1.391399171	1.607182495	−0.589310512
6	0.402815043	0.551868311	−1.107004986
8	−0.540572545	0.974724420	−1.994986782
6	−0.925938829	−2.393280675	−0.158493309
8	−0.244455719	−2.611023899	−1.181646582
7	−1.463111301	−3.391923697	0.552146140
1	−1.919756405	−3.174323767	1.427184677
6	−1.297257650	−4.801550805	0.188447144
6	−2.577959093	−0.491328990	−0.352237010
6	−3.023029077	0.889164365	0.032121148
6	−4.032946708	1.286160505	0.875346741
7	−4.039028808	2.665028468	0.827036265
6	−3.058868182	3.061049672	−0.023471395
7	−2.427274299	2.011964997	−0.518176300
1	2.106847427	1.824481836	1.474805439
1	−1.392492183	−0.974229941	1.395741629
1	2.176935788	1.848448068	−1.305887925
1	0.820463915	2.520288964	−0.401257345
1	−3.347451753	−1.209157405	−0.052005063
1	−2.489043999	−0.558153561	−1.439966531
1	−4.735905618	0.729320307	1.475477694
1	−4.671944334	3.275058398	1.326055821
1	−2.850957359	4.096103254	−0.252260818
1	6.290042255	0.139843618	−0.255995830
1	5.333016849	−1.142778582	0.541311912
1	5.988260263	0.215278818	1.481191231
1	−2.028521110	−5.391051596	0.741916797
1	−1.464004037	−4.924822196	−0.882608670
1	−0.289511931	−5.151538430	0.430942387
7	1.166456812	−0.525278979	−1.900667704
1	2.100828904	−0.664937172	−1.498854632
1	0.607060108	−1.433870933	−1.802682167
1	1.241389415	−0.226896996	−2.876372313

Geometry: TS2

Total energy: −1137.361052
Zero-point energy: 950.5377
Hydration free energy: −252.9538712

Cartesian coordinates:

6	5.623272563	0.418485158	0.225593931
6	4.308036815	1.165817215	0.293288992
8	4.222379462	2.380640788	0.180464057
7	3.172510425	0.383342921	0.470305536
1	3.314506219	−0.527758485	0.893522328
6	1.914817238	1.048244109	0.822859641
6	0.879343540	−0.038540759	1.112484754
8	0.951025788	−0.876929355	1.999445146
7	−0.147436468	0.036524410	0.187315680
1	−2.109330727	1.855351233	−0.871775883
6	−1.167022059	−1.030326571	0.248134078
6	1.282246601	1.861403027	−0.314049741
6	0.128698679	1.014933668	−0.858472182
8	−0.807631258	1.511074288	−1.542907048
6	−0.587833568	−2.346718763	−0.329390510
8	−0.363460594	−2.450348114	−1.541492002
7	−0.405486689	−3.344556539	0.554982312
1	−0.402975635	−3.101501594	1.537406670
6	0.136198834	−4.645717899	0.166494266
6	−2.510624115	−0.767259414	−0.473288915
6	−3.323042343	0.383971990	0.035003379
6	−4.462167587	0.443207818	0.795774659
7	−4.795472707	1.782931089	0.901678453
6	−3.894830916	2.520072448	0.230654912
7	−3.004827019	1.688582744	−0.295823459
1	2.054473556	1.662945509	1.720356303
1	−1.364538537	−1.163167596	1.315857948
1	1.988468956	2.158117143	−1.086843055
1	0.835983531	2.779925738	0.080465529
1	−3.103929059	−1.678349987	−0.348120895
1	−2.335537524	−0.650427341	−1.545268824
1	−5.058436350	−0.333187947	1.248290462
1	−5.594762670	2.156126782	1.397957205
1	−3.902685119	3.595409068	0.139376373
1	6.200059953	0.797254153	−0.621314671
1	5.504254752	−0.664050879	0.130944289
1	6.196301968	0.634494129	1.133356195
1	−0.195323696	−5.395871002	0.886340453
1	−0.234027270	−4.903000922	−0.826183171
1	1.231224550	−4.630302467	0.139824324
7	1.085337647	−0.056819860	−2.180062922
1	2.060148092	−0.260182110	−1.960046092
1	0.568962993	−0.937815296	−2.293456699
1	1.033716412	0.495025493	−3.034062813

Geometry: PC

Total energy: −1137.375655
Zero-point energy: 947.1483
Hydration free energy: −267.1979077

Cartesian coordinates:

6	5.684947062	1.194806743	0.714555357
6	4.276360356	1.589083450	0.329904776
8	3.991668875	2.685419049	−0.141124408
7	3.297588538	0.636268469	0.514998638
1	3.487949049	−0.189264806	1.067437754
6	1.922542251	1.004572616	0.284813412
6	0.956252222	0.019697520	0.921232739
8	1.102270660	−0.609666082	1.951837470
7	−0.192688489	−0.048747665	0.113449474
1	−2.283907956	1.703620769	−1.380855218
6	−1.295176019	−0.943243430	0.533066868
6	1.441293746	1.063719020	−1.176826538
6	0.004638390	0.617721165	−1.104294604
8	−0.874592660	0.826499771	−1.933490298
6	−0.951402477	−2.408258217	0.119587405
8	−1.293329432	−2.836063509	−0.981366491
7	−0.299594098	−3.125308856	1.057974991
1	0.078287988	−2.625331750	1.854406232
6	0.125332190	−4.505240929	0.834116746
6	−2.699774281	−0.630960782	−0.026604031
6	−3.255318191	0.736633748	0.220657559
6	−4.144345415	1.215396360	1.148019106
7	−4.362253535	2.549142424	0.845724737
6	−3.640839508	2.890048226	−0.230159857
7	−2.972326507	1.806024622	−0.615511711
1	1.738328115	1.986861691	0.747826116
1	−1.312979596	−0.867220295	1.623990322
1	1.958749415	0.310905936	−1.786108467
1	1.540657527	2.043450871	−1.644034972
1	−3.372130814	−1.354968361	0.443407930
1	−2.712794615	−0.851434226	−1.095929615
1	−4.643554589	0.726363316	1.969543760
1	−4.980011079	3.175377383	1.347577466
1	−3.610546699	3.863696078	−0.694959006
1	6.325414293	1.281316527	−0.167471140
1	5.761853125	0.180138020	1.113683524
1	6.055905862	1.905146273	1.458537941
1	0.103270020	−5.044503039	1.783127953
1	−0.563266330	−4.975197878	0.131748165
1	1.138528118	−4.551088244	0.419613125
7	1.323015265	−2.014172676	−2.647375141
1	0.453581980	−2.499113180	−2.424639980
1	2.068803079	−2.705413446	−2.604360075
1	1.256473394	−1.729152631	−3.622632562

Geometry: P

Total energy: −1080.410361
Zero-point energy: 816.3417
Hydration free energy: −81.3119773

Cartesian coordinates:

6	5.686530427	0.445161297	0.525708954
6	4.340491890	0.972273764	0.068873389
8	4.218176870	2.021655935	−0.555896885
7	3.249729070	0.195061647	0.373846090
1	3.334596188	−0.536667172	1.068736001
6	1.913738753	0.698030786	0.129281611
6	0.881958837	−0.253139153	0.722129958
8	1.007430862	−0.905771733	1.747858114
7	−0.229575813	−0.273241281	−0.106449852
1	−2.314518323	2.064889791	−1.549840468
6	−1.459424191	−0.989297574	0.304053682
6	1.473590139	0.824062424	−1.337224186
6	0.026963771	0.357184899	−1.341170223
8	−0.780050107	0.486416872	−2.238744191
6	−1.408310256	−2.467465046	−0.174067008
8	−2.129316525	−2.889818941	−1.071564012
7	−0.515698330	−3.242812964	0.492788600
6	−0.338121063	−4.651069082	0.162448787
6	−2.773232251	−0.312330459	−0.132712919
6	−2.901247527	1.125367660	0.268740223
6	−3.287442891	1.740229771	1.441542203
7	−3.260856339	3.112102514	1.315791374
6	−2.867484560	3.337645974	0.078437206
7	−2.634666755	2.172042158	−0.596020276
1	1.784655535	1.671902657	0.625837760
1	−1.410364387	−0.983856478	1.397217189
1	2.055121379	0.152914351	−1.979211999
1	1.563222495	1.832211461	−1.741903370
1	−3.573351176	−0.889984832	0.340993548
1	−2.905566460	−0.434877536	−1.208006541
1	−3.594852011	1.267208351	2.364684850
1	−2.739370613	4.308998747	−0.378708523
1	6.342833388	0.358915725	−0.344411152
1	5.628598198	−0.524215452	1.027916702
1	6.135632682	1.176023486	1.204479524
1	0.402730854	−5.077315437	0.840939111
1	−1.281387678	−5.194608962	0.270786138
1	0.004354779	−4.772642942	−0.870189233
1	0.047147050	−2.820748905	1.221514629

Geometry: NH_3

Total energy: −56.566977
Zero-point energy: 90.1855
Hydration free energy: −19.4912509

Cartesian coordinates:

7	0.000000000	0.000000000	0.108262171
1	0.949447645	0.000000000	−0.252611733
1	−0.474723823	−0.822245780	−0.252611733
1	−0.474723823	0.822245780	−0.252611733
